# Effects of Toxic and Non-Toxic *Microcystis aeruginosa* on the Defense System of *Ceratophyllum demersum*–*Scenedesmus obliquus*

**DOI:** 10.3390/microorganisms12112261

**Published:** 2024-11-08

**Authors:** Yuanpu Sha, Shuwen Zhang, Jing Dong, Xiaofei Gao, Huatao Yuan, Jingxiao Zhang, Yunni Gao, Xuejun Li

**Affiliations:** 1 College of Fisheries, Henan Normal University, Jianshe Road, Xinxiang 453007, China; 2 Observation and Research Station on Water Ecosystem in Danjiangkou Reservoir of Henan Province, Nanyang 474450, China

**Keywords:** induced defense, *Ceratophyllum demersum*, *Scenedesmus obliquus*, algal colony, MC-LR, microorganism

## Abstract

The effects of toxic and non-toxic *Microcystis aeruginosa* on the *Ceratophyllum demersum*–*Scenedesmus obliquus* system were simulated in the laboratory, and some parameters in relation to these organisms were measured. In this experiment, *C. demersum* increased the biomass of *S. obliquus*, and both toxic and non-toxic *M. aeruginosa* significantly inhibited the colony formation of *S. obliquus* and inhibited the promotion of *S. obliquus* biomass. On the 14th day, the soluble polysaccharide content of *C. demersum* decreased when it was coexisted with *S. obliquus*, but it rose again because of *M. aeruginosa*, which significantly increased the protein content of *C. demersum*. The species composition and diversity of epiphytic microorganisms also vary with different treatments. Proteobacteria is dominant in all the groups, especially in the Toxic_SMC group. In addition, bacteria that can degrade organic pollutants are more abundant in Toxic_SMC group. This study focuses on the defense response of *S. obliquus* induced by *C. demersum* under the pressure of toxic or non-toxic *M. aeruginosa* and evaluates the changes to *C. demersum* and its epiphytic microorganisms, which provides insights for the study of aquatic plant–algae integrated action systems in eutrophic or cyanobacterial blooms.

## 1. Introduction

Aquatic macrophytes play a crucial role in aquatic ecosystems, as they function as primary producers. They not only contribute to the purification of water quality and the improvement of water transparency, but also provide shelter for various organisms and impact the spatial distribution pattern of the plankton communities in water bodies [1,2]. Studies have demonstrated that certain submerged macrophytes have the ability to effectively restrain the growth of cyanobacteria, particularly *Microcystis aeruginosa*. Among the various methods available for controlling cyanobacteria blooms, biological approaches are considered environmentally friendly, simple to implement, and less likely to cause secondary pollution [3,4,5]. Currently, the widely accepted biological method primarily relies on the root absorption and allelopathy of aquatic macrophytes [6,7]. Allelopathy refers to the ability of submerged macrophytes to release chemically active substances [8], such as polyphenols, fatty acids, terpenoids, alkaloids, and more, which inhibit the growth of neighboring organisms [9,10].

In water bodies with abundant submerged macrophytes, green algae are typically the dominant species. These beneficial algae serve as primary producers in freshwater ecosystems, providing natural prey for aquatic animals and contributing to energy transmission and ecological stability [11]. Researchers have discovered that allelopathic substances secreted by submerged plants not only impact the biomass of green algae but also significantly influence their morphology and distribution [12]. For example, during periods when submerged macrophytes are prevalent in China’s Dianchi Lake, the dominant green algae species are *Scenedesmus* sp., *Pediastrum* sp., and *Coelastrum* sp. [13]. It has been reported that certain submerged macrophytes, such as *Stratiotes aloides* and *Ceratophyllum demersum*, induce the formation of colonies in *Chlorella vulgaris* and *S. obliquus* through co-cultivation, exclusion, and extraction experiments [14,15,16]. Studies have also demonstrated that *Acorus calamus*, *Pontederia cordata*, *Azolla pinnata*, and *Nymphaea* sp. promote or inhibit the growth of green algae at low or high doses [17]. The induction of colony formation in green algae by macrophytes may explain the dominance of green algae in the presence of macrophytes [15,16].

Furthermore, the multicellular communities formed by aggregated green algae often exceed the size range of zooplankton, effectively reducing their risk of being consumed. This plays a vital role in maintaining the biomass and dominance of green algae in water [18]. Currently, two widely recognized mechanisms for the formation of green algae colonies exist: one involves daughter cells remaining attached to the mother cells after reproduction [19], whereas the other involves the aggregation of single green algae cells [20]. It has been observed that increased exopolysaccharides contribute to the transition from single-cell to multi-cell morphology in green algae, as they increase the surface viscosity of the cells [15,21,22]. Despite advancements in understanding, research on the chemically active substances that induce the colony formation of green algae by submerged macrophytes remains scarce. The specific mechanisms and active components involved are still not well understood. Besides, it was observed that the ability of green algae to form colonies was not always stable. In instances in which external disturbance reduced the pressure of interspecific competition, the colonial algal cells would revert back to their unicellular forms [15,23]. The unicellular form of green algae is better suited to the size range of zooplankton and increases their feeding sensitivity, which has a significant impact on the structure of the food web and the community.

In recent years, there have been studies reporting that the colony formation of green algae induced by zooplankton or macrophytes has been destroyed due to the increased use of dissolved antibiotics or surface active agents [24,25]. The consequences of this destruction include a reduction in the dominance of green algae and changes in the structure of phytoplankton. It has also been found that heavy metals such as Cu^2+^ and Zn^2+^ can interfere with the transfer of allelochemicals along the food chain, leading to disruptions in the induced colony formation of green algae [26,27].

Cyanobacteria are a type of algae with a long history of evolution that can photosynthesize and produce oxygen. Cyanobacteria have different effects because of the variety of species. Some cyanobacteria are driven by the imbalance of nitrogen and phosphorus in water to form red tides or blooms [28,29], which seriously endangers water resources and fishery resources. Some cyanobacteria, such as *Microcystis* sp., release toxins into the environment that can poison human organs [30,31]. There are also some economically valuable cyanobacteria, such as *Nostoc Sphaeroides* and *Spirulina* sp., with high nutritional value [32,33]. In water bodies, many kinds of microorganisms coexist with cyanobacteria, but compared with other microorganisms, cyanobacteria are more dominant and competitive [34]. Therefore, the structure of the microbial network is relatively simple during the outbreak of water blooms [35], but the microbial network may be more complex and stable during the decline of water blooms than during their outbreak. At present, compared with some mature physical, chemical, and biological methods, the technology of using water bacteria to reduce cyanobacteria toxins is not mature [36].

Submerged macrophytes have the ability to inhibit the growth of cyanobacteria in eutrophic water, thereby maintaining the biomass and colonies of green algae. This regulation of the algal phase helps to shift the dominant species from cyanobacteria to green algae. However, during the degradation of *M. aeruginosa* induced by allelopathic submerged macrophytes, it has been found that the macrophytes release several times or even tens of times the normal dose of microcystins (MCs) into the water [37,38]. Additionally, phenolic acids, found in many allelochemicals, promote the expression of toxic synthesis genes (*mcyB* and *mcyD*) in toxic *M. aeruginosa*, resulting in an increase in the content of microcystins in the water [39,40]. Through co-cultivation of *Scenedesmus*, Zhu et al. (2017) [41] discovered that the presence of *Microcystis aeruginosa*. resulted in a decrease in the colonial cell number of *Scenedesmus* sp. Furthermore, as the density of *Microcystis* sp. increased, the average colonial cell number of *Scenedesmus* sp. decreased even further. However, it remains unclear whether the active substances secreted by toxic *Microcystis* can interfere with the colony formation of green algae induced by submerged macrophytes during freshwater restoration. The phenotypic plasticity of green algae and the stress response of microorganisms to cyanobacteria toxins are not clear enough, and the bloom environment of cyanobacteria may change the morphological response of green algae induced by aquatic plants. Based on this, this study explored the changes in the growth and colony formation of *S. obliquus* induced by the submerged plant *C. demersum* in the presence of *M. aeruginosa*, preliminarily explored the algal phase transformation process induced by sinking water plants in eutrophic water and predicted the algal community structure. Most importantly, it provides a scientific reference for microalgae biotechnology and the biological restoration of eutrophic water bodies.

## 2. Materials and Methods

### 2.1. Acclimation of Submerged Plants and Algae

The toxic strain of *M. aeruginosa* (FACHB-905), non-toxic strain of *M. aeruginosa* (FACHB-1005), and *S. obliquus* (FACHB-417) used in the experiment were obtained from the Freshwater Algae Collection of the Institute of Hydrobiology, Chinese Academy of Sciences, Wuhan, China. They were then acclimated in modified BG_11_ medium (1/10 BG_11_) [42]. The cultivation conditions were 25 °C with a light intensity of 25 μmol photons s^−1^ m^−2^ and a photoperiod of 12 h: 12 h [43,44].

The *C. demersum* used in this study was obtained from the aquaculture base of Henan Normal University (35° 19′ 38.363″ N, 113° 54′ 09.482″ E). All sampled macrophytes were thoroughly rinsed to remove surface contaminants and periphyton [45]. The robust apical section of the plants (10 cm in length) was obtained and acclimatized in the 1/10 BG_11_ medium. The cultivation conditions were the same as for the algae mentioned above.

### 2.2. Experimental Design

In this experiment, in order to detect the influences of toxic and non-toxic *M. aeruginosa* on the interaction between *C. demersum* and *S. obliquus*, five groups were set, as follows: (1) *C. demersum* alone (C); (2) *S. obliquus* + plastic grass (S), to remove the shading effects of the submerged macrophytes; (3) *S. obliquus + C. demersum* (SC); (4) *S. obliquus* + *C. demersum* + FACHB-905 (Toxic_SMC); (5) *S. obliquus* + *C. demersum* + FACHB-1005 (SMC). Each group was set with three parallels, and the initial biomass of *C. demersum* was 5 g/L, with the initial optical density (OD_665_) of *M. aeruginosa* at 0.1 and the initial OD_680_ of *S. obliquus* at 0.1. At the beginning of the experiment, the initial algal density of *M. aeruginosa* was 5.71 × 10^5^ cells/L, and that of *S. obliquus* was 5.95 × 10^4^ cells/L. The experimental system was cultured in 1/10 BG_11_ medium.

### 2.3. Parameter Measurement

#### 2.3.1. Growth of *S. obliquus* and MC-LR Concentration in *M. aeruginosa*

On days 0, 1, 7, and 14 of the experiment, 1 mL algal samples were obtained and counted under an optical microscope. The specific rate of growth, colony proportion (cell number ≥ 3), and average cell number per colony of *S. obliquus* in each treatment were calculated using the following formulas:

Specific rate of growth (*SGR*) = (lnN_t2_ − lnN_t1_)/(t_2_ − t_1_) (1)

Colony proportion (P_C_) = N2/N × 100% (2)

Average cells per colony (A) = ΣW × Q/ΣQ(3)

where the following apply: N_t1,_ number of cells at t_1_; N_t2_, number of cells at t_2_; N2, the cell density of *S. obliquus* with colonial morphology; N, total cell density; W, the average cell numbers of the colonial *S. obliquus*; Q, the colony numbers of *S. obliquus*.

#### 2.3.2. Determination of Soluble Proteins and Polysaccharides in *C. demersum*

A total of 0.1 g of *C. demersum* was sampled into a 1.5 mL centrifuge tube at the beginning (0 d) and end of the experiment (14 d). Next, 1 mL of 0.1 M PBS was added to homogenize the tissue. After centrifugation at 4 °C with a speed of 10,000 rpm for 10 min, the crude soluble proteins were obtained and measured using the Coomassie Brilliant Blue-G250 method [46].

For the determination of soluble polysaccharides, a sample of 0.1 g of *C. demersum* was taken and homogenized with 0.1 M PBS. The sample was then subjected to a water bath at 100 °C for 10 min and cooled to 30 °C to stop the reaction. After centrifugation, the soluble intracellular polysaccharides (IPS) were obtained and determined using the Anthrone colorimetric method [46].

#### 2.3.3. Analysis of the Epiphytic Microorganisms of *C. demersum*

At the end of the experimentation, the fresh plant tissues in each treatment were set into a sterile centrifuge, to which 10 mL of 0.1 M PBS were added, and then ultrasonically washed for 1 min. This was repeated three times [47]. The washing liquid from the three washings was collected and filtered using a 0.22 μm acetate fiber filter membrane. The membrane was stored at −80 °C in a sterile 10 mL centrifuge tube for subsequent microbial diversity detection and analysis [48]. DNA extraction, high-throughput sequencing, and analysis were performed by Shanghai Majorbio Bio-Pharm Technology Co., Ltd., Shanghai, China.

### 2.4. Statistical Analysis

All the data were obtained in triplicate. Microsoft Excel 2019 software was utilized for calculating, GraphPad Prism 10.1.2 and Origin 2021 software were used to analyze dates and plot the results, and all the data were expressed as mean ± SD. One-way analysis of variance (one-way ANOVA) was used to determine significant differences among each group. Statistical significance was set at *p* < 0.05. The microbial community diversity in the *C. demersum* biofilm was described and analyzed by Majorbio online analysis software (http://www.majorbio.com/), accessed on 18 December 2023.

## 3. Result

### 3.1. S. obliquus Abundance and Colony

**Figure 1:**
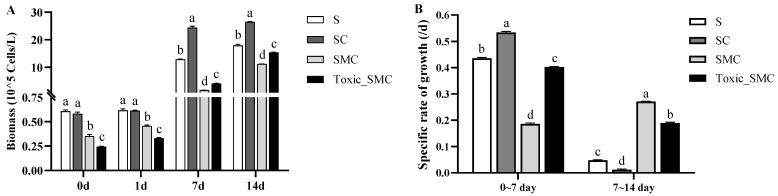
Biomass of different groups of *S. obliquus* at different times, and specific rates of growth of different groups of *S. obliquus* in different time periods. Mean values and standard deviations were calculated for the different replicates (*n* = 3); different letters represent significant differences detected (*p* < 0.05).

During the experiment, the biomass of the C group (presence of *C. demersum*) became gradually higher than that of the S group (*S. obliquus* alone) (Figure 1A). The specific cell growth rate of *S. obliquus* was significantly higher in the presence of *C. demersum* (SC group) compared to the control with *S. obliquus* alone (S group) from the 0th to the 7th day. However, from the 7th to the 14th day, the growth rate was significantly lower in the SC group than in the other three groups. Similarly, the specific growth rate of *S. obliquus* in the presence of toxic *M. aeruginosa* (Toxic_SMC group) was higher from the 0th to the 7th day but lower than that with the non-toxic *M. aeruginosa* (SMC group) from the 7th to the 14th day (*p* < 0.05) (Figure 1B).

**Figure 2:**
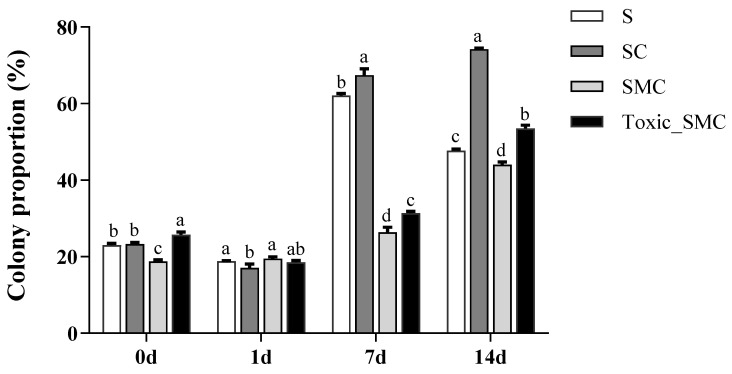
Colony proportion of different groups of *S. obliquus* in different time periods. Mean values and standard deviations were calculated for the different replicates (*n* = 3); different letters represent significant differences detected (*p* < 0.05).

**Figure 3:**
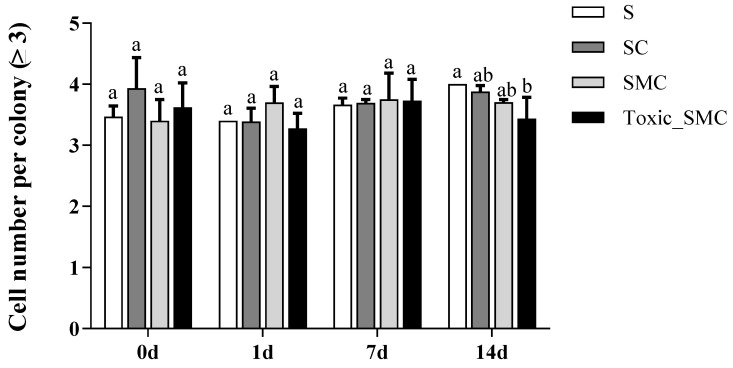
Cell number per colony (≥3) of different groups of *S. obliquus* in different time periods. Mean values and standard deviations were calculated for the different replicates (*n* = 3); different letters represent significant differences detected (*p* < 0.05).

Furthermore, the presence of *C. demersum* significantly promoted the colony formation of *S. obliquus*, whereas the presence of both toxic and non-toxic *M. aeruginosa* significantly reduced colony induction. This reduction was observed as a decrease in the colony proportion of *S. obliquus* in the SMC and Toxic_SMC groups. It should be noted that the colony proportion was lower in the SMC group compared to the Toxic_SMC group (*p* < 0.05) (Figure 2). However, in the present experiment, there was no significant difference in the cell number per colony among the experimental groups in different periods (*p* > 0.05), except that the Toxic_SMC group had significantly fewer cells per colony than the S group on the 14th day (*p* < 0.05) (Figure 3). The ANOVA table also showed that the experimental treatment and date had significant effects on the biomass and colony proportion of *S. obliquus*, but for the average cell number per colony, the experimental conditions made almost no difference (Table 1).

### 3.2. Soluble Proteins and Polysaccharides of C. demersum

**Figure 4:**
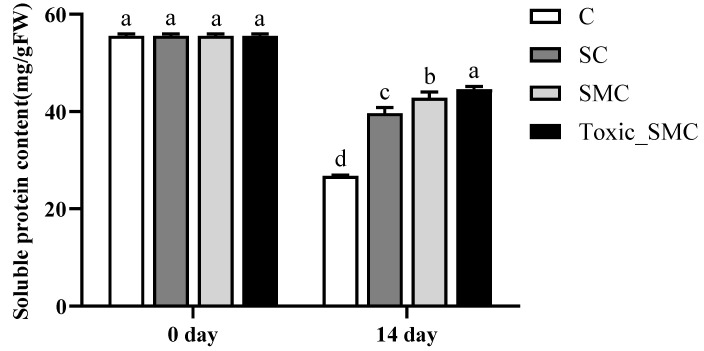
The soluble protein contents in each group on the 0th day and 14th day of the experiment. Mean values and standard deviations were calculated for the different replicates (*n* = 3); different letters represent significant differences detected (*p* < 0.05).

At the end of the experiment, on the 14th day, the soluble protein content of *C. demersum* (group C) was the lowest, and it was higher when co-cultivated with *S. obliquus*. Furthermore, the protein content of *C. demersum* significantly increased after the addition of *M. aeruginosa*, with toxic *M. aeruginosa* showing a more pronounced effect compared to non-toxic *M. aeruginosa* (*p* < 0.05) (Figure 4).

**Figure 5:**
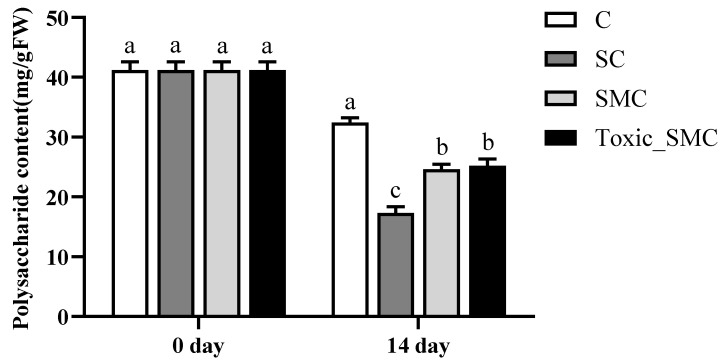
The soluble polysaccharide contents in each group on the 0th day and 14th day of the experiment. Mean values and standard deviations were calculated for the different replicates (*n* = 3); different letters represent significant differences detected (*p* < 0.05).

In addition, at the end of the experiment, the soluble polysaccharide content in *C. demersum* (group C) was the highest, but it significantly decreased when co-cultured with *S. obliquus* (*p* < 0.05). The presence of *M. aeruginosa* significantly improved the soluble polysaccharide content in the SC group, with no significant differences observed between toxic and non-toxic *M. aeruginosa* (Figure 5).

### 3.3. Analysis of Epiphytic Microorganisms in C. demersum

**Figure 6:**
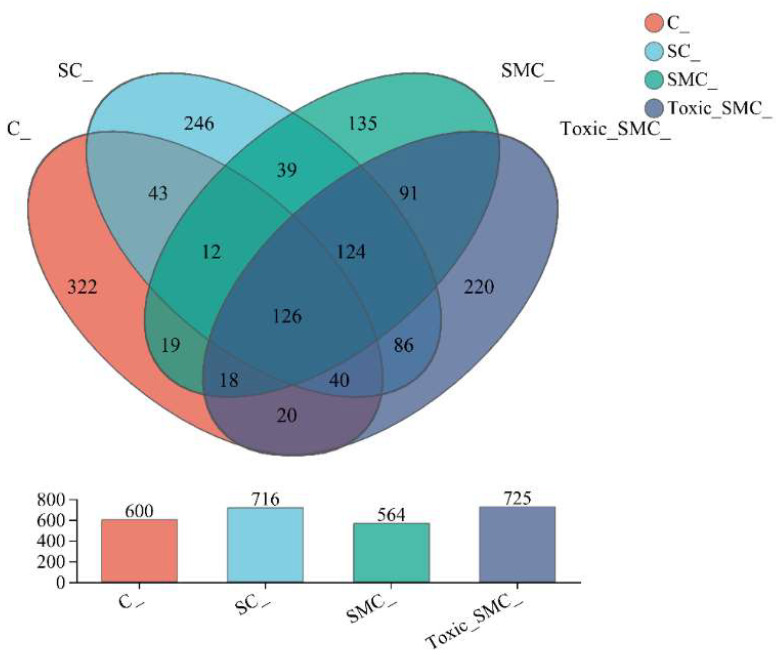
Microbial community analysis: Venn diagram; C: *C. demersum*; EU: *C. demersum* and *S. obliquus* are co-cultured; DU: *C. demersum*, *S. obliquus*, and non-toxic *M. aeruginosa* are co-cultured; DU: *C. demersum*, *S. obliquus*, and toxic *M. aeruginosa* are co-cultured.

**Figure 7:**
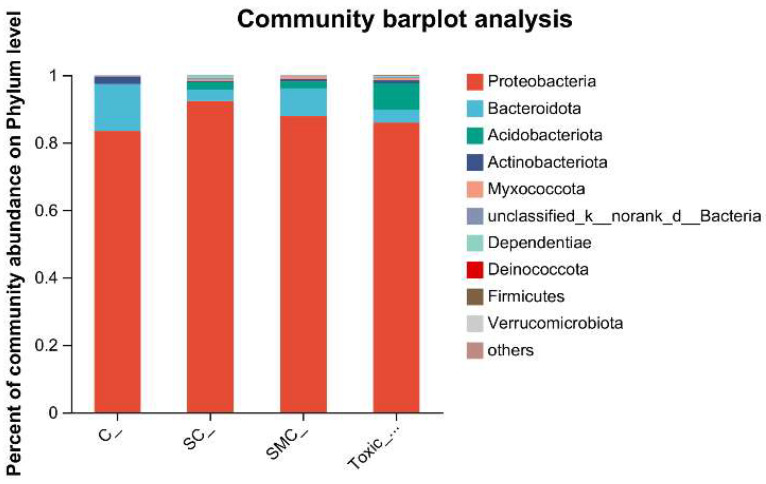
Microbial community analysis: percentage community abundance of phylum.

**Figure 8:**
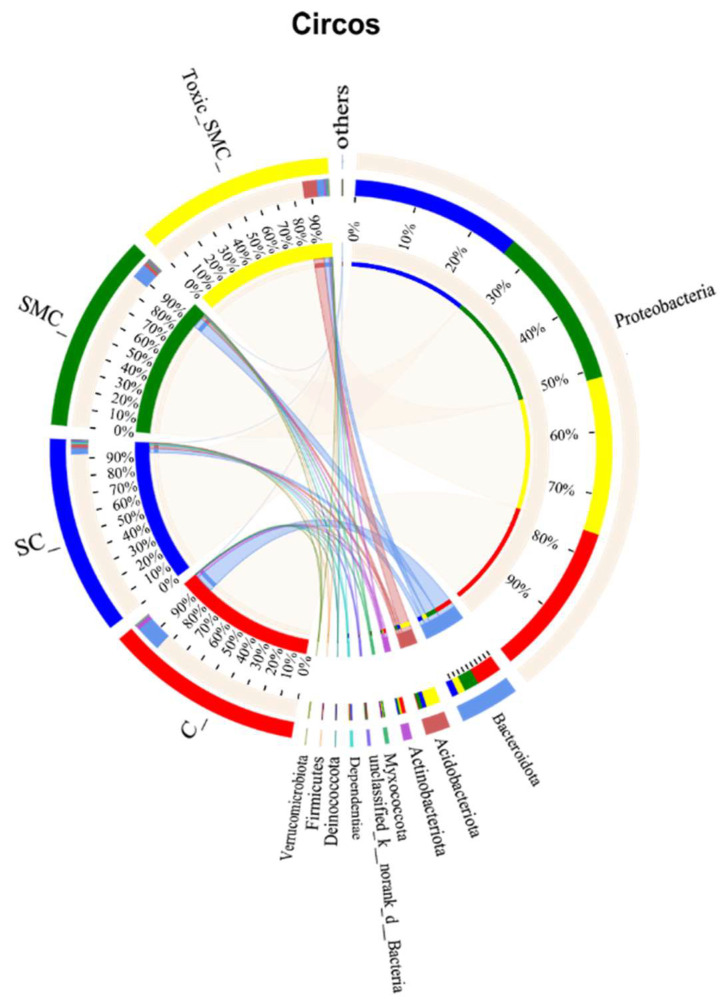
Microbial community analysis: Circos plot.

The Venn diagram in Figure 6 shows that the unique OTU values were highest in the C group and lowest in the SMC group, with 322 (C), 246 (SC), 135 (SMC), and 220 (Toxic_SMC) unique OTUs observed. The Circos diagram and column diagram depicting the percentage accumulation of bacterial community at the phylum level revealed that Proteobacteria was the dominant phylum, with an abundance of over 80% in each treatment. Additionally, Bacteroidetes and Actinobacteriota were the most abundant in the group of *C. demersum* cultivation alone. Acidobacteriota and Myxococcota were found in the co-cultivation of *C. demersum* and *S. obliquus*, with their abundance being highest in the Toxic_SMC group (Figure 7 and Figure 8).

**Figure 9:**
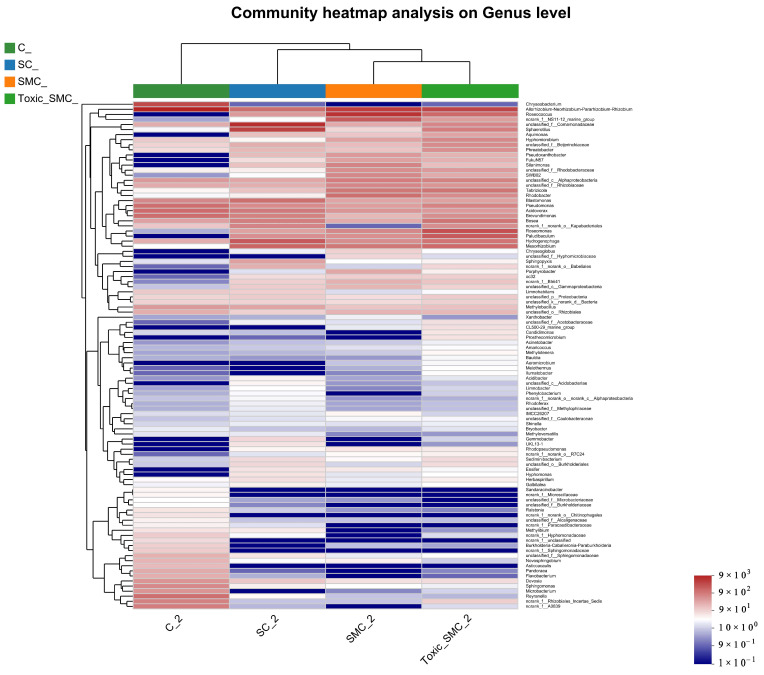
Microbial structure at genus level: heatmap of genus levels in different samples.

The cluster heatmap in Figure 9 displays the top 100 genera at the genera level. Group C stands out, with two major bacterial genera, *Chryseobacterium* and *Allorhizobium–Neorhizobium–Pararhizobium*, dominated by *C. demersum* alone. Additionally, *Sandaracinobacter*, *Asticcacaulis*, and *Microbacterium* were more prevalent in group C compared to the other three groups. Conversely, the abundance of *Hyphomonas* in the SC, SMC, and Toxic_SMC groups significantly increased in comparison to the cultivation of *C. demersum* alone. Moreover, the SMC group exhibited significantly lower abundances of *Candidimonas* and *Prosthecomicrobium* when compared to the Toxic_SMC group (Figure 9).

## 4. Discussion

### 4.1. Growth and Morphology Changes of S. obliquus

The morphological changes induced by submerged macrophytes are a defense strategy employed by green algae when confronted with adverse conditions. These changes promote colony formation, leading to increased sedimentation rates, reduced interspecific competition among photosynthetic organisms, and the prevention of predation. Consequently, green algae can maintain their dominance in aquatic ecosystems [49,50]. In this study, it was further demonstrated that *C. demersum* can enhance the colony formation of *S. obliquus*. This finding aligns with previous research that has shown the promoting effects of *C. demersum* and *Elodea densa* on *S. obliquus* and C. vulgaris colony formation through co-cultivation, extract application, and simulation experiments [51,52]. Meanwhile, the presence of *M. aeruginosa* in the experimental system greatly disrupted the induced colony formation of *S. obliquus* by *C. demersum*. There are two possible explanations for this. Firstly, cyanobacteria are more sensitive to allelochemicals compared to green algae [53], which means that the chemical signals produced by *C. demersum* may have been transmitted more effectively to *M. aeruginosa* than to *S. obliquus*. In the process of transmission, the chemical information substances produced diversion, and the decrease in the allelochemicals accepted by *S. obliquus* resulted in a decrease in the population proportion. Secondly, the toxicity of MC-LR could have caused physiological damage to *C. demersum* [54], thereby interfering with the secretion of chemically active substances by aquatic macrophytes. Previous study has been reported that MC-LR can increase the soluble polysaccharide content in and out of *S. obliquus* cells [55], which may explain why the colony proportion of *S. obliquus* was higher in the Toxic_SMC group compared to the SMC group in our study. Additionally, the average cell number per colony of *S. obliquus* in the Toxic_SMC group was slightly lower than that in the SMC group on the 1st, 7th, and 14th days of the experiment, indicating that the active substances secreted by toxic *M. aeruginosa* could inhibit the formation of larger colonies by *S. obliquus*.

In the present study, the cell abundance of *S. obliquus* in the SC group was significantly higher than that in the S group. This finding is consistent with El-Darier et al.’s observations (2021) [56], suggesting that the allelopathic effects of *C. demersum* on *S. obliquus* exhibit a dose-dependent promotion and inhibition pattern. The possible reason for this is that the response of *S. obliquus* to allelopathy is in dose-dependent promotion and inhibition mode. Some green algae also have a similar phenomenon in response to allelopathy from other organisms [56], that is, a low concentration of allelopathy can promote growth, while a high concentration of allelopathy can even inhibit growth. At the same time, the interspecific competition, including nutrient salts and light, as well as the interaction of chemical active substances, will affect the growth of *S. obliquus*, and *M. aeruginosa* has a competitive advantage over *S. obliquus* [57]. When green algae and *M. aeruginosa* co-existed, MC-LR released by the latter caused damage to the photosynthetic system, and activated the protective mechanism of the photosynthetic system by reducing the concentration of chlorophyll a and carotenoid, triggering oxidative stress and inhibiting the growth of green algae [58]. Moreover, MC-LR can slow down the growth of *S. obliquus* [59].

### 4.2. Soluble Proteins and Polysaccharides of C. demersum

Photosynthetic organisms produce ATP and NADPH in the light reaction stage of photosynthesis, so that H_2_O and CO_2_ can be synthesized into compounds in the dark reaction stage and stable chemical energy can be obtained to generate photosynthetic products [60]. There will be interspecific competition between *C. demersum* and *Scenedesmus* in co-culture. A decrease in photosynthesis makes *C. demersum* produce fewer photosynthetic products than when it grows alone, and its monosaccharide production is bound to be affected. At the same time, *S. obliquus* forms a population due to allelopathy, and this phenotypic defense is related to the soluble polysaccharide level of *S. obliquus* [15,22], which requires *S. obliquus* to increase the productivity of soluble polysaccharide. The decrease in raw materials for polysaccharide production in the environment makes the distribution of soluble photosynthetic products of *C. demersum* tilt towards protein. Under stress, such as exposure to microcystins, large plants can increase their soluble protein and polysaccharide synthesis as one of their defensive measures [54], and at the same time, *S. obliquus* changes from the colony to the single cell phenotype, which reduces the demand and competition of polysaccharide synthesis raw materials, which can also affect the recovery of the soluble polysaccharide content of *C. demersum*.

### 4.3. Epiphytic Microorganism of C. demersum

The results from the Venn diagram indicate significant changes in the species composition of the microbial community in each experimental group compared to the control group. The highest abundance of OTUs was observed in the Toxic_SMC group, suggesting that toxic *M. aeruginosa* has an impact on the diversity of biofilm microbial communities. Furthermore, the bacterial community of *C. demersum* varied at the phylum and genus levels across different treatments. Proteobacteria, which are commonly found in freshwater environments and attached bacterial communities of aquatic macrophytes [61,62,63], were the dominant phyla in all the experimental groups. The presence of Proteobacteria is known to enhance the degradation of cyanobacterial toxins, potentially contributing to the control of cyanobacteria by *C. demersum* [64]. The *Allorhizobium–Neorhizobium–Pararhizobium–Rhizobium* bacteria primarily utilize ammonia as their nitrogen source [65]. The abundance of this genus remained consistently high across all the experimental groups, possibly due to the low oxygen content in the subaqueous environment. Actinobacteriota, which typically inhabit water with low nutrient concentrations, were most abundant in the *C. demersum*-alone cultivation. Bacteria belonging to the phylum Bacteroides, such as *Flavobacterium*, are known for their efficient hydrocarbon degradation capabilities and are commonly found in a range of aerobic and anaerobic environments, where they degrade carbohydrates [66]. The higher abundance of Bacteroides in the *C. demersum*-alone cultivation compared to the other treatment groups may be attributed to the higher polysaccharide content in this group. The abundance of *Hyphomonas* increased in the presence of toxic *M. aeruginosa*, suggesting its potential role as an organic pollutant and toxin-degrading bacterium. *Sandaracinobacter*, which contains chlorophyll a [67], was not dominant in the co-culture system of *C. demersum* and *S. obliquus*, where competition for photosynthesis is intensified. *Asticcacaulis* and *Microbacterium* are aerobic and chemoheterotrophic bacteria that are more likely to thrive in low-competition environments, and their occurrence was not suitable in the water body where the MCs were produced. *Candidimonas*, known for its ability to degrade organic pollutants [68], showed higher abundance in the Toxic_SMC group compared to the SMC group, which may be attributed to the higher concentration of MC-LR in the Toxic_SMC group.

## 5. Conclusions

In this study, the effects of *M. aeruginosa* on the formation of *S. obliquus* colonies induced by *C. demersum*, and the response of the *C. demersu*–*S. obliquus* system to *M. aeruginosa* were investigated. The growth of *S. obliquus* was promoted under the influence of *C demersum*, but it was inhibited under the influence of *M. aeruginosa*. *M. aeruginosa* threatened the *C. demersum*-induced colony formation of *S. obliquus*, reduced colony proportion, improved the grazing sensitivity of *S. obliquus*, and restored the interspecific competition between *C. demersum* and *S. obliquus*. The soluble polysaccharide synthesis and soluble protein production of *C. demersum* were increased under the stimulation of declustering and *M. aeruginosa*. The response of epiphytic microorganisms in *C. demersum* to MCs is evident in a change in community species diversity. The predominance of Proteobacteria remained unchanged, and the diversity of Bacteroidetes decreased significantly with the decrease in soluble polysaccharide content in *C. demersum*. The survival of aerobic chemoheterotrophic bacteria such as *Asticcacaulis* and *Microbacterium* was threatened by MCs, and their diversity decreased. Under MCs exposure, the species diversity of organic-degrading bacteria such as *Hyphomonas* and *Candidimonas* increased. These changes to the attached microorganisms of *C. demersum* tend to adapt to and alleviate the environmental conditions stressed by *M. aeruginosa*.

## Figures and Tables

**Table 1:** Repeated measurement analysis of variance comparing algal biomass, colony proportion, and cell number per colony under different experiment conditions during the experimental period.

Source of Variation	Biomass		Colony Proportion		Cell Number Per Colony	
	*F*	*p*	*F*	*p*	*F*	*p*
Treatment	6658	*p* < 0.0001	1216	*p* < 0.0001	1.829	0.2834
Day	25,144	*p* < 0.0001	5293	*p* < 0.0001	3.246	0.1750
Treatment × Day	2743	*p* < 0.0001	781.8	*p* < 0.0001	1.598	0.3208

## Data Availability

The data that support the findings of this study are available from the corresponding author upon reasonable request.
